# Use of Fluoxetine in Treating Compulsive Sexual Behavior: A Case Report

**DOI:** 10.7759/cureus.29245

**Published:** 2022-09-16

**Authors:** Gurraj Singh, Harsehaj Singh, Sherlyne Magny, Inderpreet Virk, Manpreet Gill

**Affiliations:** 1 Psychiatry, Sri Guru Ram Das Institute of Medical Sciences and Research, Amritsar, IND; 2 Medicine, American University of Integrative Sciences, School of Medicine, St. Michael, BRB; 3 Psychiatry, California Department of Corrections and Rehabilitation, Sacramento, USA; 4 Psychiatry and Behavioral Sciences, Interfaith Medical Center, New York City, USA

**Keywords:** selective serotonin reuptake inhibitor (ssri), sex addiction, fluoxetine, sexual disorders, compulsive sexual behavior

## Abstract

Compulsive sexual behavior (CSB) disorder is generally characterized by recurrent and intense sexually arousing fantasies, sexual urges, and behaviors that cause individual distress or impair daily functioning. CSB has significant consequences, including but not limited to personal distress, depression, anxiety, and a high risk of sexually transmitted diseases. CSB is often seen along with other psychiatric disorders, most commonly with major depressive and substance use disorders. A primary goal of treatment for CSB is to help manage the uncontrollable impulses and urges a patient endures by reducing excessive sexual behavior with the use of psychotherapy, self-help groups, and medications such as selective serotonin reuptake inhibitors (SSRIs). SSRIs are well-known for their pharmacotherapeutic role in many psychiatric and medical conditions; however, symptoms of hyposexuality are notable side effects. Here we report our findings on a patient, a 36-year-old male who classified himself as a sex addict since late adolescence, participating in various forms of sexual-related activities at high frequency. He presented to the outpatient clinic for treatment for depression alongside his CSB. He was successfully treated with an SSRI, fluoxetine.

## Introduction

The term compulsive sexual behavior (CSB) is synonymously and interchangeably used with terms such as hypersexuality, sex addiction, and nymphomania. It is defined as difficulty in controlling excessive or inappropriate sexual urges, cravings, fantasies, or behavior [1]. With an estimated 3-6% prevalence in the common population and starting in late adolescence or early adulthood [2], those who exhibit CSB experience repetitive and intrusive sexual thoughts that bring about distress to individuals or impair daily functioning [1]. Furthermore, this disruption is often seen to increase with time [3]. CSB can be categorized under the following forms of behavior; paraphilic and nonparaphilic. Paraphilic CSB is behavior that is deemed socially unacceptable that can involve non-human objects, the suffering of one’s self or a partner, children, or a non-consenting person (i.e., fetishism, exhibitionism, and pedophilia) [4]. In contrast, nonparaphilic CSB is depicted by more typical sexual desires, which include compulsive sexual acts and multiple partners within the parameters of consent [5]. Men and women experience CSB differently, with higher reported incidences amongst males than females [3]. Males are more likely to exhibit compulsive behavior toward pornography, masturbation, casual, anonymous sex with strangers, paid sex, and sex with multiple partners [1]. However, a higher frequency of masturbation, number of sexual partners, and pornography are seen amongst females [6]. Association of CSB with other comorbidities can often be observed. For example, a study reported that 100% of the sampled group presented with a psychiatric disorder, most commonly being major depressive disorder (58%) and sexual dysfunction (46%) [7]. In addition, a prevalence of substance use disorder (34-71%) and impulse control disorder (i.e., pathological gambling 9.4-30.9%) were also identified as potential comorbidities with CSB [8]. Fluoxetine, a selective serotonin reuptake inhibitor (SSRI), is used in treating a wide array of disorders but is commonly prescribed as an antidepressant. One of the common side effects seen during routine fluoxetine use is drug-induced sexual dysfunction, with a decrease in sexual desire and sexual function [9]. This inadvertently can be prescribed as a pharmacotherapeutic option for reducing CSB. Despite a lack of definitive accepted definition outlined in the Diagnostic and Statistical Manual of Mental Disorders, Fifth Edition (DSM-5), evidence indicates CSB can be commonly seen in the adult population and can be a source of significant personal distress. The aim of this case report is to review the clinical aspects presented in CSB and propose treatment approaches that can be pursued in the management of this disorder.

## Case presentation

Mr. X, a 36-year-old homosexual male, single, domiciled, and self-employed, presented to the outpatient clinic with complaints of feeling distressed related to his ongoing sexual behavior and practices that first started in his late teens. He endorsed a history of having strong compulsive sexual urges, thoughts, and preoccupations that would lead him to engage in high-risk sexual behavior and activities. Some of his high-risk sexual behavior involved participating in “hooking up” with male strangers at random, watching pornography excessively, engaging in group sex, multiple orgies, and attending swinger clubs with multiple male partners simultaneously. The patient claimed that for years, engaging in countless sexual acts with men, some without protection. The patient was reported taking Descovy (emtricitabine/tenofovir) for HIV prophylaxis, despite his HIV status being seronegative. He reported no known history of any childhood trauma, victimization, or fixations. Unfortunately, no additional history from a reliable informant could be received because he tends to stay isolated and did not give consent to get further information from familial history. Previously, the patient had been in individual and group psychotherapy and participated in many sex addiction support groups but did not find them helpful. Mr. X also endorsed a history of recreational substance use and reported feeling “low and depressed” for the last two months. No additional rating scales or screening questionnaires were used in follow-up for depression. His substance use consisted of a recreational pattern involving snorting cocaine 4-5 times a year, ketamine 2-3 times/month, occasional alcohol use, rare spontaneous use of ecstasy, and smoking marijuana 2-3 times/week. According to the patient, he stopped doing drugs because he felt they would keep him awake and prevent him from getting sleep. However, the patient continued to engage in high-risk sexual behavior even during times of sobriety. At the time of the initial visit, he denied any drug-seeking behavior, history of admissions to detox or rehabs, or any legal issues related to his substance use. With the stoppage of drug use, the patient had been experiencing significant dysphoric mood and poor energy levels and was unable to feel a “full range of emotion” and “muted.” At the time of first clinical contact, he denied any issues related to sleep or appetite and denied suicidal or homicidal ideations, intentions, or any plan. There was no history of sexual abuse, with no suggestive history of mania, hypomania, anxiety, obsessive-compulsive disorder, or psychotic disorder. There was no history of seizure disorder, focal neurologic deficit, or head injury. There was no family history of any psychiatric disorder. All routine blood investigations were within normal limits, and urine drug screen revealed no abnormality. After discussing various medication options with the patient, he agreed to a trial of fluoxetine to help with his mood and CSB. He was started on fluoxetine 20 mg a day which was titrated to 40 mg a day for four weeks. An increase in dosage was tried up to 60 mg a day, but the patient experienced moderate nausea and requested to keep the dose at 40 mg. Over a consistent period of 6-8 weeks, a gradual improvement in his overall symptoms was observed. The patient reported a significant change with an elevated “better” mood and energy levels and a noticeable reduction in his sexual urges. After about four months of treatment, the patient expressed continued satisfaction and effectiveness in his current fluoxetine regimen with no change in dosage. He described a reduction of symptoms to almost 80-90%, along with improved day-to-day functioning. The patient continued to experience compulsiveness on fewer occasions, with no eliciting triggers, as described by the patient. This improvement has been maintained and ongoing since the last follow-up at six months. Psychotherapy was recommended, but the patient declined. He instead enrolled himself in Men’s Queer groups with regular attendance.

## Discussion

In the field of research, there is a clear indication of the limited management efforts of CSB. Many reasons include problems selecting treatment options for individuals with this disorder and the absence of consistent diagnostic criteria [10]. The agreed guidelines, however, for diagnosing CSB are existing sexual urges or behaviors that are difficult to control [10]. The patient discussed here is a case who presented with severe distress due to a strong desire to participate in sexual-related behaviors. Further probing showed that he also has a history of depression and substance abuse.

Disorders with similar CSB symptoms should be examined to determine if they commonly coexist. Substance use disorder presents symptoms similar to CSB, such as urges, time-consumed behaviors, triggers, and strong desires. A study has shown that being male and meeting the criteria for cocaine abuse and dependency were significantly associated with hypersexuality [11]. Due to overlapping features along with shared dopaminergic and serotonergic pathways, research has argued whether CSB should also be considered an addiction. These studies suggest that disorders with shared CSB symptoms and neuropathways should be screened for excessive sexual patterns and characteristics. It is also necessary to consider treatments that are effective in addressing conditions that co-occur with hypersexual behaviors.

The practice of sexual-related behaviors or excessive sexual fantasies may be due to underlying rationales or conditions such as a history of trauma and depression. In this case, the patient has a history of depression and cyclic substance use, with no known history of childhood trauma, victimization, or fixations. CSB may play a mechanistic coping role whenever the condition(s) is triggered or experienced. Developing this condition may allow individuals to adjust to stressful events or difficult emotions. Hence, post-traumatic stress disorder and complex post-traumatic stress disorder can display a significant direct effect on hypersexual behavior [12]. In addition, depression due to past traumatic events can act as a significant indirect mediator of trauma-hypersexual behavior relations [12]. However, without treatment, the underlying disorder(s) may continue and increase psychosexual behaviors. These conditions can cause severe distress in the individual.

Excessive practices such as masturbation and pornography may cause physical and emotional impairments and disturbances in daily functioning. Excessive sexual acts have been related to negative consequences such as work-related problems, personal problems, relationship problems, and risky behavior [13]. Sexual compulsivity also increases the risk of sexually transmitted infections due to risky practices. Thus, educating individuals on the use of preventative measures and screenings for sexually transmitted infections should be prioritized.

Drug treatments like fluoxetine, an SSRI, proved to be effective for hypersexual symptoms, especially in individuals with existing depression. Fluoxetine is a well-known antidepressant that increases serotonin in the brain to improve mood, sleep, and energy. However, research suggests these undesirable secondary effects in serotonergic medications may be useful for treating hypersexuality in individuals, which further supports the use of SSRIs for our patient [14]. No additional side effects of fluoxetine were observed or expressed by the patient. The medication may lessen and manage excessive sexual-related behaviors in individuals diagnosed with CSB. Medications that act as SSRIs are currently being studied in individuals with CSB and have shown to be effective [10]. The patient with a history of depression and a pattern of substance use responded well to oral fluoxetine and is recommended to initiate psychotherapy. Impulse control behavior, obsessive-compulsive behavior, and other mood symptoms can contribute to CSB. However, these were not entirely ruled out. 

Medications besides SSRIs, such as opiate antagonists and mood stabilizers, have been used to treat CSB [15]. Selecting specific medications to target and treat conditions that coexist with CSB should be considered. Naltrexone, an opiate antagonist, was shown to be effective for an individual with a co-occurring disorder when SSRIs failed. Treating the underlying condition(s) with specific medications that also manage urges and cravings or alter sexual hormone function are strategies that may be useful.

## Conclusions

This case highlights the importance of determining effective treatments, such as using SSRIs for individuals with CSB. In addition to medications, implementing psychosocial treatments and programs such as support groups and therapies can help identify core triggers and experiences in individuals with CSB. As a result, these individuals will be able to build self-awareness, develop positive sexual behaviors, and lessen distress. Additionally, clinicians should be aware that CSB often overlaps with various disorders such as depression. Hence, screening for co-occurring disorders is equally essential when considering effective drugs and treatments. Implementing screenings for co-occurring disorders will enhance the identification of CSB and determine specific effective treatment options.
